# Accurate classification of secondary progression in multiple sclerosis using a decision tree

**DOI:** 10.1177/1352458520975323

**Published:** 2020-12-02

**Authors:** Ryan Ramanujam, Feng Zhu, Katharina Fink, Virginija Danylaitė Karrenbauer, Johannes Lorscheider, Pascal Benkert, Elaine Kingwell, Helen Tremlett, Jan Hillert, Ali Manouchehrinia, The BeAMS Study group

**Affiliations:** Department of Clinical Neuroscience, Karolinska Institutet, Stockholm, Sweden/Department of Mathematics, KTH—Royal Institute of Technology, Stockholm, Sweden; Faculty of Medicine (Neurology), UBC Hospital, and Djavad Mowafaghian Centre for Brain Health, University of British Columbia, Vancouver, BC, Canada; Department of Clinical Neuroscience, Karolinska Institutet, Stockholm, Sweden/Neuro Theme, Karolinska University Hospital, Stockholm, Sweden/Academic Specialist Center, Multiple Sclerosis Centre, Stockholm, Sweden; Department of Clinical Neuroscience, Karolinska Institutet, Stockholm, Sweden/Neuro Theme, Karolinska University Hospital, Stockholm, Sweden; Neurologic Clinic and Policlinic, Departments of Medicine and Clinical Research, University Hospital Basel, University of Basel, Basel, Switzerland; Clinical Trial Unit, Department of Clinical Research, University Hospital Basel, Basel, Switzerland; Faculty of Medicine (Neurology), UBC Hospital, and Djavad Mowafaghian Centre for Brain Health, University of British Columbia, Vancouver, BC, Canada; Faculty of Medicine (Neurology), UBC Hospital, and Djavad Mowafaghian Centre for Brain Health, University of British Columbia, Vancouver, BC, Canada; Department of Clinical Neuroscience, Karolinska Institutet, Stockholm, Sweden/Neuro Theme, Karolinska University Hospital, Stockholm, Sweden; Department of Clinical Neuroscience, Karolinska Institutet, Stockholm, Sweden/The Karolinska Neuroimmunology & Multiple Sclerosis Centre, Centre for Molecular Medicine (CMM), Karolinska Institutet, Stockholm, Sweden

**Keywords:** Multiple sclerosis, classification, secondary progressive, decision tree

## Abstract

**Background:**

The absence of reliable imaging or biological markers of phenotype transition in multiple sclerosis (MS) makes assignment of current phenotype status difficult.

**Objective:**

The authors sought to determine whether clinical information can be used to accurately assign current disease phenotypes.

**Methods:**

Data from the clinical visits of 14,387 MS patients in Sweden were collected. Classifying algorithms based on several demographic and clinical factors were examined. Results obtained from the best classifier when predicting neurologist recorded disease classification were replicated in an independent cohort from British Columbia and were compared to a previously published algorithm and clinical judgment of three neurologists.

**Results:**

A decision tree (the classifier) containing only most recently available expanded disability scale status score and age obtained 89.3% (95% confidence intervals (CIs): 88.8–89.8) classification accuracy, defined as concordance with the latest reported status. Validation in the independent cohort resulted in 82.0% (95% CI: 81.0–83.1) accuracy. A previously published classification algorithm with slight modifications achieved 77.8% (95% CI: 77.1–78.4) accuracy. With complete patient history of 100 patients, three neurologists obtained 84.3% accuracy compared with 85% for the classifier using the same data.

**Conclusion:**

The classifier can be used to standardize definitions of disease phenotype across different cohorts. Clinically, this model could assist neurologists by providing additional information.

## Introduction

Multiple sclerosis (MS) is a chronic demyelinating disorder, most often of a relapsing–remitting (RR) course. After many years, the disease course typically converts to a secondary progressive (SP) phase, wherein accumulation of irreversible disability occurs and the disease progresses steadily throughout a patient’s remaining life, often in the absence of clinical relapses.^1^ The average time from an RR disease onset to transition to SP disease is approximately 20 years.^2^ There are important clinical implications when a patient has reached SPMS, since most disease-modifying drugs (DMDs) are indicated during the RR phase of MS.^3^ DMDs’ efficacies also appear to wane as a person ages and the SP phase is reached.^4^

The most common method of assessing the time at which the patient has transitioned to the SP phase is a retrospective clinical review of a patient’s medical history, including the expanded disability status scale (EDSS) scores^5^ over time. However, this approach may vary among clinicians or countries with different assessment criteria. Furthermore, neurologists may feel hesitant to make such an irreversible determination early or at the time of transition, as an assignment of an SP course may render patients with limited DMD options. An objective measure of transition to SPMS that relies on basic clinical measurements would potentially benefit both researchers and clinicians.^6^ In clinical research, such a tool could create a uniform basis for unbiased classification, thereby minimizing variation between and within studies. This tool could also benefit in the clinic by providing a complimentary metric to assist in decision-making and reinforce clinical assessment.

We used a large pool of patients with known disease phenotype and basic clinical variables in order to build and verify a classifier. We included validation from an independent cohort and comparisons to existing methods of assigning SP disease status.

## Materials and methods

### Patient materials

MS patients with an RR disease course at MS symptom onset (RR-onset) and available information on date of birth, date of MS symptom onset, sex, year of SP transition (if applicable) and the date and score of the most recent EDSS (*n* = 14,387) were extracted from the Swedish MS registry (SMSreg, hereafter referred to as the “Swedish cohort”).^7^ For the Swedish cohort, the SP transition date is assigned retrospectively by the attending neurologist during a clinical visit based on international consensus criteria.^8^ The cohort was used to build the classifier.

A cohort of 5431 RR-onset MS patients from British Columbia, Canada (hereafter referred to as the “Canadian cohort”) was used to validate the classifier. This cohort has been previously described^9,10^ and was selected because similar information was available, including the assignment of the SP transition date.

## Construction of decision tree classifier

Several types of machine learning classification methods including support vector machines, random forest, and logistic regression model were tested with available data. Relative accuracies of each technique are shown in Table 1. Decision trees were ultimately selected as they generate very clear rules which are easy to interpret and can be readily applied in clinical practice.^11^ When assessing a patient’s clinical course, transparency to the underlying model decisions is preferred, since the relevant factors can be easily confirmed manually. To benchmark the decision tree results, an alternative model was created by logistic regression using the same data as the final decision tree. Logistic regression was chosen due to ease of use, interpretability of results, and scaling via the logit function from 0 to 1.

**Table 1.** Accuracy of classifiers of SPMS, including sensitivity, specificity, positive predictive value (PPV^a^) and negative predictive value (NPV).

Classifier (and cohort)	*N*	Accuracy (%)	Sensitivity (%)	Specificity (%)	PPV (%)	NPV (%)
Decision trees (Swedish cohort)	14,387	89.3	93.7	79.9	90.1	85.4
Decision trees (Canadian cohort validation)	5,431	82.0	89.8	71.4	81.2	83.5
MSBase algorithm (Swedish cohort)	14,387	77.8	76.6	85.5	97.2	35.9
Logistic regression (Swedish cohort)	14,387	89.3	94.0	79.2	90.1	86.0
Random forest (Swedish cohort)	14,387	89.3	93.6	80.1	91.0	85.4
Support vector machine (Swedish cohort)	14,387	88.6	93.6	77.7	90.0	85.0
Neurologists (averaged, Swedish cohort)^b^	100	84.3	92.8	53.2	88.0	66.7

a RR assigned as positive class.

b Average accuracy of three neurologists examining full records of 100 patients. The decision tree model classified 85 of these 100 correctly by comparison.

The recursive partitioning (rPART) method^11^ was used to identify the optimal split of the data that would best classify the patients into the two phenotypes—RR and SP. In the first instance, five fully grown decision tree classifiers with no limit on the complexity parameter were developed using combinations of age at the most recently available EDSS assessment, EDSS score, sex, age at MS symptom onset, and disease duration (from symptom onset) at the EDSS assessment. Accuracy of these trees was compared and variables that did not affect the classification accuracy (e.g. sex) or deemed replaceable by a more accessible variable (e.g. age instead of disease duration) were then removed to simplify the final tree. Remaining variables in the final model included only age and the EDSS assessment, both at the latest clinical visit. The fully grown decision tree classifier based on these variables was then pruned (post-pruning) to its simplest state by setting the complexity parameter to that of the tree with the smallest cross-validation error (complexity parameter = 0.0001). The complexity parameter is the minimum improvement in the model needed in each node. Complexity parameter controls the tree growth and prevent overfitting. In short, by setting the complexity parameter to 0.0001, we pruned off any split in the tree that did not improve the fit and reduced the size of the tree from 96 to 9 splits.

We then calculated the accuracy, sensitivity, specificity, positive predictive value (PPV), and negative predictive value (NPV) of the decision tree classifier for predicting disease phenotype (RR vs. SP) at the time of the most recently available EDSS score.

### Comparison with MSBase SP algorithm

A comparison with an existing method of estimating disease status was conducted.^12^ Derived from data extracted from the MSBase registry, a large international observational MS collaboration, this algorithm is based on longitudinal data for each patient. The MSBase algorithm assigns conversion to SPMS if the following criteria are met: At least a one-point increase in the EDSS for patients with an EDSS <6, and at least a 0.5-point increase in patients with an EDSS ⩾6, in the absence of a clinical relapse. In addition, an EDSS ⩾4 must be reached, and a pyramidal functional system (FS) score of 2 or above, both confirmed at a second visit at least 3 months later (confirmed EDSS progression). In the original work,^12^ this definition achieved 87% diagnostic accuracy (compared with a consensus diagnosis of three MS neurologists) and was able to detect SPMS more than 3 years earlier than the physicians’ clinical assessment (using information from the same database). In this study, this algorithm was adapted to ignore the FS scores criterion (due to lack of availability in our data). Based on relative accuracies of the various models generated in the MSBase algorithm, we expect that this adaptation should have a minimal effect on score accuracy. Furthermore, many MS clinical databases worldwide do not routinely collect the FS sub-scores.

### Comparison with clinical evaluations

Three MS neurologists from the Karolinska University Hospital, Sweden (K.F., J.H., and V.D.), independently and blindly reviewed the clinical records from 100 randomly chosen patients with RR-onset to determine how clinical assessments compared with decision tree classifier. In the first instance, two of these neurologists classified patients using only the variables at the latest visit which were included in the decision tree classifier. Then, all three neurologists repeated the classifications by using complete patient clinical records including all recorded patient visits with EDSS scores, relapses, and so on.

### Comparison of time to SP conversion between different methods of classification

To compare average rates of conversion to SPMS between the different methods of estimating disease status, Kaplan–Meier plots were utilized with the time to SP assessed from birth as well as from MS symptom onset. The tree classifier outputs constructed here and predictions from the MSBase SP algorithm^12^ were used and compared to the phenotype labels assigned by neurologists in the registry.

The software that was used to analyze data included R version 3.2.3^13^ and the packages “e1071,” “party,” “rpart,” “rpart.plot,” and “partykit.” Ethical permission for the study was granted by the Stockholm Regional Ethical Committee and the University of British Columbia’s Clinical Research Ethics Board.

#### Data availability

The Swedish data related to the current article are available from Jan Hillert, Karolinska Institutet. To be able to share data from the Swedish MS registry, a data transfer agreement along with appropriate ethical permissions need to be obtained between Karolinska Institutet and the institution requesting data access. This is in accordance with the data protection legislation in Europe (General Data Protection Regulation (GDPR)). Persons interested in obtaining access to the data should contact Ali Manouchehrinia (ali.manouchehrinia@ki.se).

## Results

### Study population

In total, 14,387 patients were included in the Swedish cohort of which 71.8% were female; the average age at onset of MS was 32.4 years (standard deviation (SD) ± 10.2). Mean age at the most recent MS clinic visit with an EDSS score was 48.6 years (SD ± 12.9) and median disease duration was 14.0 years (interquartile range (IQR): 7.0–23.0). By the date of data extraction (February 2019), 68% of the patients remained in the RR phase and 32% had transitioned to SPMS (Table 2).

**Table 2.** Characteristics of the Swedish and Canadian cohorts used to build and externally validate the decision tree classifier.

	Swedish cohort			Canadian cohort
	Remained in the relapsing–remitting phase at the most recent clinic visit (*n* = 9,830)	Reached secondary progressive phase at the most recent clinic visit (*n* = 4,557)	All (*n* = 14,387)	All (*n* = 5,431)
Age at the most recent clinic visit mean (SD) (years)	44.0 (11.5)	58.4 (9.7)	48.6 (12.9)	47.0 (11.3)
Sex (female%)	7056 (71.8%)	3211 (70.5%)	10,267 (71.4%)	4432 (74.0%)
Multiple sclerosis symptom onset age mean (SD) (years)	32.1 (10.0)	33.0 (10.5)	32.4 (10.2)	31.6 (9.6)
Disease duration at the most recent clinic visit (years) (median [IQR])	10.0 [5.0–17.0]	25.0 [17.0–33.0]	14.0 [7.0–23.0]	14.0 [7.0–22.0]
Most recent EDSS score (median [IQR])	1.5 [1.0–2.5]	6.5 [4.5–7.5]	2.5 [1.0–5.0]	3.5 [1.5–5.5]

EDSS: expanded disability status scale, IQR: interquartile range, SD: standard deviation.

### Decision tree classifier

**Figure 1.**
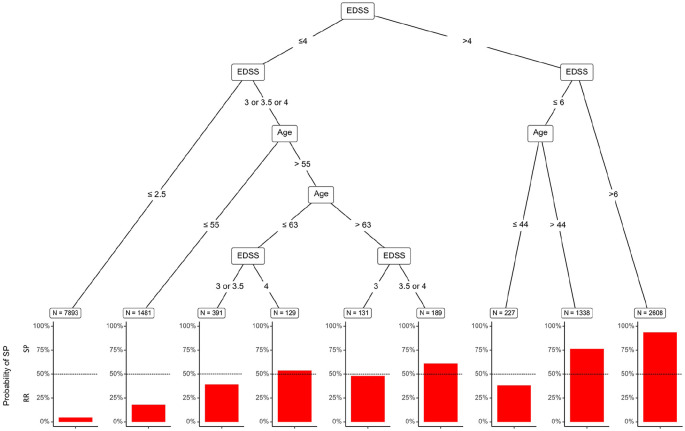
Pruned decision tree classifier based on a MS patient’s age and EDSS score. Terminal nodes indicate the number of individuals and the bar length indicates the probability of SPMS.

**Figure 2.**
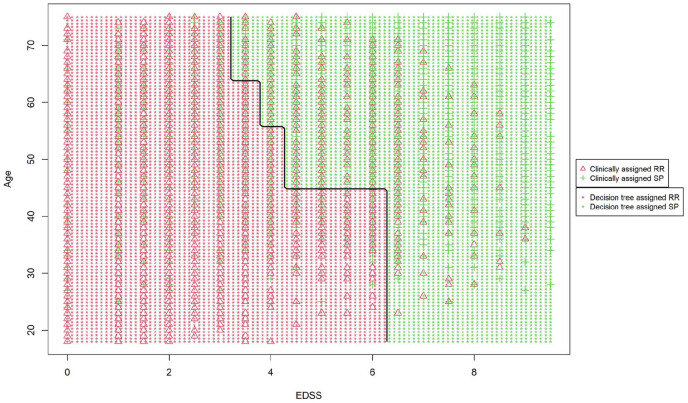
Decision boundaries of the decision tree relative to the EDSS score and the age at the latest assessment.

**Figure 3.**
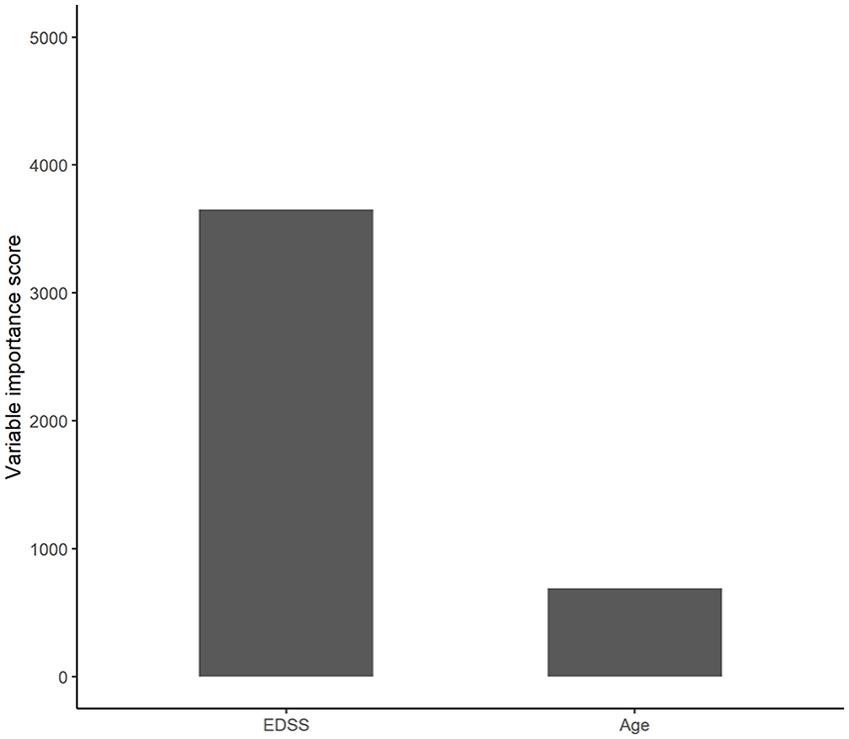
Variable importance plot generated in the decision tree indicating the relative importance of the two predictor variables.

All decision tree classifiers yielded similar accuracies ranging from 89.5% (95% confidence intervals (CIs): 89.1–90.1) for the model containing the last EDSS score, age at last visit, sex, and disease duration to 89.3% (95% CI: 88.8–89.8) for the model containing only the last EDSS score and age at last visit. Given the simplicity of the latter and the similar accuracy between models, the model using most recently available EDSS score and age was chosen as the final decision tree classifier (Table 3 and Figure 1). Figure 2 presents the decision boundaries of the decision tree. Variables’ importance scores generated in the decision tree are presented in Figure 3.

**Table 3.** Full decision tree model and corresponding terminal node probabilities of SPMS.

SPMS probability	Classification	EDSS	Age (years)
0.04	RR	<3	Any
0.18	RR	3 or 3.5 or 4	<56
0.38	RR	4.5 or 5 or 5.5 or 6	<45
0.39	RR	3 or 3.5	56–64
0.48	RR	3	⩾64
0.53	SP	4	56–64
0.61	SP	3.5 or 4	⩾64
0.76	SP	4.5 or 5 or 5.5 or 6	⩾45
0.93	SP	>6	Any

EDSS: expanded disability status scale, SP: secondary progressive, SPMS: secondary progressive multiple sclerosis. RR: relapsing–remitting.

### Internal and external validation

The internal accuracy of the decision tree model, when constructed and tested on the Swedish cohort, was 89.3% (95% CI: 88.8–89.8). The Canadian cohort included 5431 relapsing-onset patients of whom 1954 (36%) had transitioned to SP by the end of follow-up. Mean age at the end of follow-up was 47 years (SD ± 11.3) and median last available EDSS score was 3.5 (IQR: 4). When tested for validation accuracy in the Canadian cohort, the model was 82.0% (95% CI: 81.0–83.1) accurate at determining the clinically assigned disease phenotype by an MS neurologist (Table 1).

### Comparisons to MSBase algorithm

The MSBase algorithm achieved 77.8% (95% CI: 77.1–78.4) classification accuracy when applied to the Swedish cohort. The MSBase algorithm is more conservative in assigning SPMS and achieved higher specificity and subsequently higher PPV as compared with the decision tree classifier (Table 1). Characteristics of the patients misclassified by the decision tree and the MSBase algorithm as compared to the clinically assigned phenotype in the Swedish cohort are presented in Table 4. RR patients misclassified as SP in both approaches were generally older, with longer disease duration and higher EDSS scores at the most recent clinic visit. SP patients misclassified as RR by decision trees had significantly lower EDSS scores compared to clinically assigned SP patients. Misclassification of SP patients by the MSBase algorithm was mainly due to the absence of confirmed progression.

**Table 4.** Characteristics of patients misclassified by the decision tree classifier and MSBase algorithm.

	Clinically assigned RR in the Swedish cohort			Clinically assigned SP in the Swedish cohort		
	Clinically assigned RR (reference phenotype) (*n* = 9,830)	Misclassified to SP		Clinically assigned SP (reference phenotype) (*n* = 4,557)	Misclassified to RR	
		Decision tree classifier (*n* = 622)	MSBase algorithm (*n* = 278)		Decision tree classifier (*n* = 915)	MSBase algorithm (*n* = 2,921)
**Age at the most recent clinic visit (mean (SD)) (years)**	44.0 (11.5)	55.7 (9.7)	50.1 (11.3)	58.4 (9.6)	53.5 (10.4)	58.0 (9.8)
**Sex (female%)**	7056 (71.8%)	457 (73.5%)	196 (70.5%)	3211 (70.5%)	660 (72.1%)	2069 (70.8%)
**Multiple sclerosis symptom onset age (mean (SD)) (years)**	32.1 (10.0)	37.4 (11.5)	33.8 (11.1)	33.0 (10.5)	33.8 (10.6)	33.4 (10.6)
**Disease duration at the most recent clinic visit (years) (median [IQR])**	10.0 [5.0–17.0]	17.0 [10.0, 25.0]	14.0 [9.0, 21.0]	25.0 [17.0–33.0]	18.0 [12.0, 25.5]	23.0 [16.0, 32.0]
**Most recent EDSS score (median [IQR])**	1.5 [1.0–2.5]	5.5 [4.5, 6.5]	5.0 [4.0, 6.0]	6.5 [4.5–7.5]	3.0 [2.0, 3.5]	6.0 [3.5, 7.0]

EDSS: expanded disability status scale, IQR: interquartile range, SD: standard deviation. RR: relapsing–remitting. SP: secondary progressive.

### Comparison to clinical evaluations by neurologists

Clinical evaluations by two neurologists on a randomly selected set of 100 patients when using only the most recent EDSS score and age were 79.0% and 87.0% accurate. The decision tree classifier was 85.0% accurate for these patients. Clinical evaluation of the same set of 100 patients but with complete clinical history by three neurologists had classification accuracies of 85.0%, 83.0%, and 85.0% (average: 84.3%; Table 1). The intraclass correlation coefficient for agreement between the three neurologists was 0.80 (95% CI: 0.73–0.86).

### Median time to SP conversion

**Figure 4.**
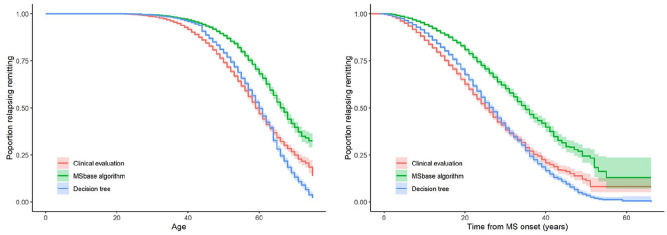
Kaplan–Meier estimate and 95% confidence intervals (colored bands) of the various models based on age (left) and time from MS onset (right) in years at transition to SPMS (total RR-onset population *n* = 13,712).

From Kaplan–Meier curves, the median time to SP from birth; that is, the age at which SP was reached was 60.1 (95% CI: 59.7–60.5) years for the decision tree classifier, 66.0 (95% CI: 65.4–66.8) years for the MSBase algorithm, and 59.3 (95% CI: 58.8–59.7) years based on the clinical evaluations for the Swedish cohort (Figure 4). From Kaplan–Meier curves, the median time to SP from MS symptom onset was 26.3 (95% CI: 25.9–26.8) years for the decision tree classifier, 34.5 (95% CI: 33.6–35.6) years for the MSBase algorithm, and 25.0 (95% CI: 24.5–25.5) years based on the clinical evaluation.

## Discussion

An accurate measurement of the probability of a patient having reached SP phase of MS could have great benefits for both clinical research and decision-making in clinical settings, especially given the hope that more DMD options will be available to manage or delay SP phase of MS. The model presented uses a decision tree classifier to obtain highly accurate estimation of current clinical course, using only the patient’s most recent EDSS score and corresponding age.

Our decision tree classifier provides an objective assessment of MS phenotype (RR or SP). This may benefit multi-site studies, including multinational clinical trials because, at present, the determination of SP phase may vary between participating centers. Generally, applying the model to assign MS phenotypes may be less prone to personal or cultural biases when assigning SP status; however, certain limitations of the EDSS, such as emphasis on motility and less emphasis on cognitive decline, may still carry forward to this model. The model may also be of value to identify patients in need of more careful clinical evaluations or when clinical history is not available. Furthermore, model-based methods allow the identification of large and homogeneous pools of data which can be used internationally, similar to the MS severity score (MSSS) and age-related multiple sclerosis severity (ARMSS) scores.^14^

## MSBase algorithm comparison

Authors of a 2016 study proposed an EDSS-based objective measure of SP phase transition for an earlier and more consistent identification of SP patients (the MSBase algorithm).^12^ Although this proposed algorithm may increase the sensitivity and specificity of SP classification as compared to clinical evaluation and can also result in more consistent phenotype assignment, the method still relies on access to longitudinally collected data, including the FS sub-scores. Our current proposed decision tree classifier, which does not rely on longitudinal data, may therefore offer an alternative approach to phenotype determination in research studies since patients who are assumed to still be in the RR phase, but have not yet been reviewed clinically, can be accurately classified. As our classifier requires access to only limited amounts of clinical data, it may also have the benefit of increasing the pool of patients potentially eligible for analyses or inclusion in a study. However, reliance only on cross-sectional data may increase the misclassification rate, possibly more so for patients with a more stable disease. Consequently, the decision tree classifier yielded lower specificity than the MSBase algorithm which uses longitudinal data. The decision tree classifier incorrectly classified 622 of 9830 RR (6.3%) patients as SP due to over reliance on cross-sectional data. These patients were on average 5 years older and had a significantly higher EDSS score (median: 5.5 vs. 1.5) at the time of their most recent assessment than the “general” RR population who were determined to still be in the RR phase by the treating neurologist.

## Comparison with neurologists

Ideally, our classifier, which is a form of supervised machine learning, could be compared against another objective measure of SPMS onset (e.g. a reliable bio- or imaging-marker). In the absence of such a marker, we compared to a neurologist-determined disease course which may be rather subjective. Clinical assignment of phenotype in our Swedish cohort is based on the collective contribution of hundreds of neurologists who typically follow their patients throughout their lives. Hence, a reasonably consistent and accurate classification of phenotypes by practicing neurologists for each of their patients is expected. This is despite the fact that each neurologist may classify their patients slightly differently with respect to EDSS and RR/SP status. A model trained on thousands of patients with different neurologists recording assessments may better generalize the differences than a single neurologist, resulting in increased consistency and accuracy. This may partly explain the lower than expected accuracy of classification by three neurologists in this work.

## Algorithm usage

Although the model has high classification accuracy, caution must be exercised when interpreting an individual patient’s status in a clinical setting. For an individual patient, classifying their disease as having progressed to the SP stage may be unsettling, as it can denote an irreversible decline in a patient’s underlying disease. Furthermore, this can trigger a discussion on disease-modifying therapy (DMT) discontinuation, as many DMTs have limited effect on the disease course at SP phase. However, with potential for newly emerging DMT options in the treatment of SPMS,^15–17^ this would likely mitigate DMT cessation and instead inform a potential treatment switch. Still, the potential for incorrectly assigning SP exists due to both the algorithm accuracy and the fact that the underlying neurologist assigned disease classification is itself subjective in nature. Therefore, this algorithm should be considered an additional data point that could be a useful addition during a clinical visit. More high probability classification might help with the neurologist’s decision-making in clinical settings regarding prognosis and treatment. In addition, the decision tree classifier can serve as a marker to notify if the assigned RR course needs to be carefully revised.

Similar to the MSBase algorithm that showed lower accuracies in the Swedish cohort than the original cohort,^12^ the accuracy of our decision tree classifier was expectedly slightly lower when applied to the Canadian cohort. This can be due to range of factors including the different time periods between the Swedish and Canadian data (Canada being a more historical dataset), differences in DMT availability during the different time periods, and differences in phenotype assignment during the different time points.

Nevertheless, the decision tree model constitutes an improvement based on not only improved accuracy but also the extremely simple data requirements for which classification can be easily determined for patients during each clinical visit, as opposed to requiring clinical assessments over time for evidence of progression independent of relapse and confirmatory EDSS scores. Simplicity of the decision tree facilitates its clinical and research utility.
